# Genomes of novel *Serratia* strains from suburban soil

**DOI:** 10.1128/mra.00866-24

**Published:** 2024-12-04

**Authors:** E. X. Markert, Landon Severe, Kaeson Severe, Katrina I. Twing, L. M. Ward

**Affiliations:** 1 Department of Geosciences, Smith College, Northampton, Massachusetts, USA; 2 Department of Microbiology, Weber State University, Ogden, Utah, USA; Wellesley College Department of Biological Sciences, Wellesley, Massachusetts, USA

**Keywords:** antibiotic resistance, horizontal gene transfer, lateral gene transfer, soil microbiology, comparative genomics

## Abstract

Here, we present genomes of three strains of *Serratia* initially isolated from suburban soil—one strain of *S. ureilytica* and two strains of *S. quinivorans*—resistant to multiple classes of antibiotics. This expands the genomic sampling of a group relevant to the ecosystem and human health.

## ANNOUNCEMENT

The genus *Serratia* has a wide distribution spanning soil, water, and host-associated environments and includes some members known to function as opportunistic pathogens in humans and other organisms (1, 2). Because of the frequency of opportunistic infections in hospital settings, the distribution and evolution of antibiotic resistance in *Serratia* are of particular concern (e.g. 3, 4). Following methods outlined in (5) for the culturing of tetracycline-resistant bacteria from natural environments, we isolated three novel pure cultures (3A-UT, 3B-UT, and 3C-UT) from a suburban yard near Ogden, Utah, on MacConkey agar infused with tetracycline (6). Via the culture-based Kirby–Bauer disk diffusion susceptibility analysis (7, 8), they were shown to be resistant to antibiotics including tetracycline, ampicillin, and erythromycin (6). DNA was extracted from the isolates using the Qiagen DNeasy PowerSoil Pro Kit following the manufacturer’s instructions (6). Additional details of the culturing and laboratory methods can be found in (6). Here, we report genome sequences of these novel strains.

Genomes were sequenced by the Utah Public Health Laboratory via 2 × 251-bp Illumina MiSeq following preparation with the Illumina Nextera DNA Flex library prep kit following the manufacturer’s instructions. Genome assembly was performed with SPAdes v. 4.0.0 (9). Genomes were analyzed as described in (10), including preliminary annotation with RAST v2.0 (11), taxonomic identification with GTDB-Tk v2.4.0 (12), and metabolic pathway presence confirmation via MetaPOAP v1.0 (13). Additional annotation was performed with Alien Hunter v1.1.0 (14) and mobileOG-db v1.1.3 (15) in Proksee (16). The final, publicly available genome was annotated with PGAP (17). Completeness and contamination were determined with CheckM v1.2.2 (18). A concatenated ribosomal protein phylogeny was constructed using RAxML v8.2.12 (19) after aligning sequences with MUSCLE v5.0.0 (20). Visualization was performed with iTOL v6.9.1 (21) after calculating transfer branch support with BOOSTER v0.1.9 (22). Presence of antibiotic resistance genes was determined with Resistance Gene Identifier v6.0.3 (23). Default parameters were used for all software. Sequence statistics for the three genomes can be found in Table 1.

**TABLE 1:** Sequence statistics

Value	3A-UT	3B-UT	3C-UT
SRA accession	SRR28911882	SRR28911881	SRR28911880
WGS accession	JBFQXS000000000	JBFQXR000000000	JBFQXQ000000000
Raw reads	3,489,606	2,781,446	7,928,580
Genome size	5,306,791	5,540,662	5,549,599
Contigs	197	74	216
N50	571,272	1,437,015	3,311,166
GC%	59.5	55.0	55.1
Coding sequences	5,265	5,255	5,320
Completeness	99.86	100	100
Coverage	141	120	336

**Fig 1:**
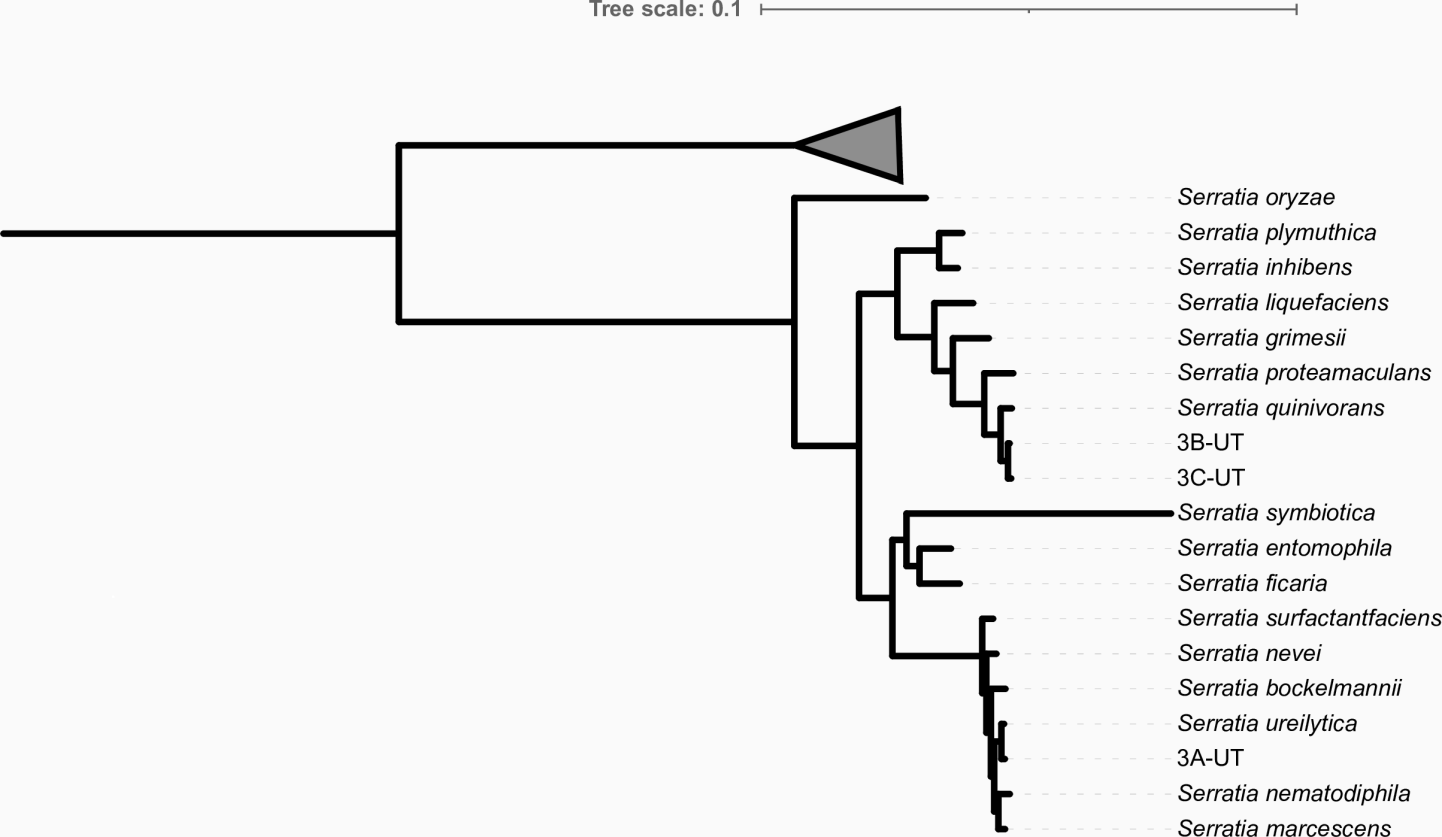
Concatenated ribosomal protein tree built using our genomes and other complete *Serratia* genomes from the NCBI, covering all recognized species in this genus according to GTDB. Rooted with members of the Enterobacteriaceae genus *Shimwellia*.

Strain 3A-UT was determined by GTDB-Tk and a concatenated ribosomal protein phylogeny (Fig. 1) to be a member of *S. ureilytica*. Strains 3B-UT and 3C-UT were determined to be members of *S. quinivorans*. Each of the three genomes encoded ~30 successfully annotated genes that were not present in conspecific reference genomes; these genes are good candidates for having undergone recent horizontal gene transfer. Each genome also encodes a large number of hypothetical proteins, particularly in 3A-UT where ~ 65% of all reads have no matches in the RefSeq database. Among potentially newly transferred annotated genes are a large number of phage-associated genes and PFGI-1 mobile genomic islands, demonstrating active transfer of the genetic material into these organisms. In addition to antibiotic resistances encoded by closely related *Serratia* strains, 3C-UT encodes carbapenem resistance via an SPR beta-lactamase. To our knowledge, this is the first observation of resistance to carbapenem in a strain of *S. quinivorans*, a concerning discovery given that carbapenem antibiotics are typically reserved to treat multidrug-resistant infections (e.g. 24).

## Data Availability

All data is publicly available through the NCBI SRA and WGS databases under accession numbers SRR28911882 and JBFQXS000000000 (3A-UT), SRR28911881 and JBFQXR000000000 (3B-UT), and SRR28911880 and JBFQXQ000000000 (3C-UT), respectively.
